# Evaluation of quality of life and physical activity in patients with type 1 diabetes mellitus during the COVID-19 pandemic

**DOI:** 10.20945/2359-3997000000531

**Published:** 2022-12-01

**Authors:** Zeliha Çelik, Füsun Baloş Törüner, Meral Boşnak Güçlü

**Affiliations:** 1 Gazi University Faculty of Health Sciences Department of Physiotherapy and Rehabilitation Ankara Turkey Department of Physiotherapy and Rehabilitation, Faculty of Health Sciences, Gazi University, Ankara, Turkey; 2 Gazi University Faculty of Medicine Department of Endocrinology and Metabolism Ankara Turkey Department of Endocrinology and Metabolism, Faculty of Medicine, Gazi University, Ankara, Turkey

**Keywords:** COVID-19, quality of life, physical activity, mental health, hypoglycemia

## Abstract

**Objective:**

The aim of the study is to compare the quality of life, physical activity, anxiety, depression, fear of hypoglycemia, loneliness perception in patients with type 1 diabetes mellitus and controls.

**Subjects and methods:**

Forty-four patients and 63 controls were included in this cross-sectional study. Quality of life (Short Form 36-SF-36), physical activity level (International Physical Activity Questionnaire-short form), anxiety and depression (Hospital Anxiety and Depression Scale), fear of hypoglycemia (Hypoglycemia Fear Survey), loneliness perception (UCLA Loneliness Scale) were evaluated.

**Results:**

Physical role limitations and general health perception subscale scores of SF-36 questionnaire in patients were significantly higher than the controls (p < 0.05).

**Conclusion:**

Role limitations due to physical problems and fear of hypoglycemia are increased, and general health perception is impaired in patients with type 1 diabetes mellitus. Physical inactivity is an important symptom in individuals in the pandemic period. In this regard, telerehabilitation approaches will be beneficial for all individuals in increasing physical activity, improving quality of life, and decreasing anxiety, depression and loneliness perception during the pandemic period for all individuals. The importance of a multidisciplinary approach in diabetes management and dealing with problems should be considered in pandemic.

## INTRODUCTION

In December 2019, a novel type coronavirus, identified as Severe Acute Respiratory Syndrome Coronavirus 2 (SARS-CoV-2) or (2019-nCoV) of unknown origin, appeared in Hubei province, China and started to spread rapidly all over the world ( 1 ). Studies reported that the epidemic disease named coronavirus disease-19 (COVID-19) causes common symptoms such as fever, cough, dyspnea, fatigue, weakness, respiratory distress, muscle pain, sore throat, loss of taste and smell ( 2 , 3 ). To prevent the spread of COVID-19, most of the countries have taken precautions like lockdown and countrywide restrictions due to increasing cases ( 4 ). Thus, unexpected quarantine measures have induced change in the lifestyle of the individuals ( 5 ). Scientific investigations have emphasized that quarantine and prolonged stay at home lead to physical inactivity, psychological problems such as anxiety, depression, loneliness and impaired quality of life ( 5 , 6 ). On the other hand, between SARS and Angiotensin Converting Enzyme-2 binding in pancreatic islets triggers damage of pancreatic islet causing to acute diabetes ( 7 ). Considering that COVID-19 and chronic diseases increase the level of inflammatory markers, the combined effect of these pathologies may worsen outcomes of patients with type 1 diabetes mellitus suffered from COVID-19 ( 8 ).

Regular physical activity and exercise are necessary to reduce negative effects of the disease in patients with chronic diseases like diabetes mellitus, hypertension ( 9 ). All of the changes in diet and exercise management of patients with diabetes mellitus related to lockdown may cause worsening health and psychological status and glycemic control ( 10 ). Previous studies emphasize that physical activity and exercise training reduces levels of HbA1c and improves glycemic control ( 11 , 12 ). However, the data obtained from multidimensional studies on the status of patients with type 1 diabetes during the pandemic are not much compared to healthy controls. Therefore, the effect of the COVID-19 pandemic on these parameters is not yet known. The aim of this study is to further evaluate the quality of life, physical activity, anxiety, depression, fear of hypoglycemia, loneliness perception of patients with type 1 diabetes mellitus compared with healthy controls in the period of COVID-19 pandemic.

## SUBJECTS AND METHODS

### Study design and participants

Patients with type 1 diabetes mellitus who were under medical treatment at Gazi University Department of Endocrinology and Metabolism and age-gender matched healthy controls were included in the study. The survey was conducted on volunteer patients who came to the hospital or called with a video interview for routine control between September and December 2020 and healthy controls without any chronic disease. Patients diagnosed with type 1 diabetes mellitus and individuals willing to participate in the study were included. Individuals who have a cognitive disorder, are unwilling to participate in the study, are not literate, and does not have sufficient knowledge and functional levels to fill out the online form were excluded from the study. Compliance with the inclusion and exclusion criteria was evaluated with the pre-interview before questionnaire by researchers. This cross-sectional study was approved (No: 2020-467/08.09.2020) by Gazi University Ethics Committee and performed following the Declaration of Helsinki. A clinical trial number was obtained (NCT04558645). All participants approved the digital informed consent form before the study. Primary outcomes were quality of life and physical activity, and secondary outcomes were anxiety, depression, fear of hypoglycemia, loneliness perception. All of the participants replied to questions in the online survey prepared via Google Forms. The demographic characteristics and clinical information such as HbA1c of the patients were obtained from the clinical files recorded in last routine controls.

### Quality of life

Quality of life was evaluated using the Turkish validated version of Short Form-36 (SF-36) health survey questionnaire ( 13 ). The scale assesses health related quality of life and includes 8 dimensions consisting of physical functioning (10 items), physical role limitations (4 items), bodily pain (2 items), general health perceptions (5 items), vitality/energy (4 items), social functioning (2 items), emotional role limitations (3 items), and mental health (5 items). Each dimension is scored from 0 (worst health) to 100 (best health) ( 14 ).

### Physical activity level

The physical activity level was evaluated using the Turkish short version of the International Physical Activity Questionnaire (IPAQ) to estimate the intensity of the physical activity of the previous week ( 15 ). The self-administered IPAQ short form contains 7 questions and provides information about time spent during vigorous activities, moderate activities, walking and sitting. The total score is obtained by summing and calculating metabolic equivalent of task (METs) values corresponding to each activity. Sitting time is not included in the total score ( 16 ). According to total scores, patients’ being physically active were classified into three groups as high (>3000 MET-min/week), moderate (600-3000 MET-min/week) and low <600 MET-min/week) physically active ( 17 ).

### Anxiety and depression

Anxiety and depression were assessed using the Hospital Anxiety and Depression Scale. The scale with fourteen items includes two subscales consisting of anxiety and depression subscales. Each item is scored from 0 to 3 points and scores vary between 0-21 points for each of the Depression and Anxiety subscales. Higher scores show higher severity. Cut off point for the anxiety subscale is 10, while depression is 7 ( 18 ).

### Fear of hypoglycemia

Fear of hypoglycemia was evaluated using the Hypoglycemia Fear Survey (HFS) ( 19 ). This survey consists of 33 items and two subgroups as behavior (HFS-B-15 items) and worry (HFS-W-18 items). The individuals answer items about their anxiety related to hypoglycemia and what they have done to prevent hypoglycemia in the past 6 months. Each item was scored from 0 to 4 points. Higher scores indicate higher fear of hypoglycemia ( 20 ) and any score >50% was shown as an indicator of fear of hypoglycemia ( 21 ).

### Loneliness perception

To evaluate the severity of loneliness perception, short form of UCLA Loneliness Scale (ULS-8) was performed. This scale includes 8 items and each item is scored between 0-4 points, items 3 and 6 are reverse coded. The sum of the 8 items gives the total loneliness score. Higher scores indicate increased severity of loneliness ( 22 , 23 ).

### Statistical analysis

In this study, at least 35 participants for each group were calculated, based on the pilot results of this study using the general health perception subscale scores of SF-36 for 0.50 effect size and 80% power (G*Power 3.0.10 system, Franz Faul, Universität Kiel, Germany) ( 24 ). The Windows-based SPSS 20 statistical analysis program was used for the analyses. Normal distribution of data was tested using ‘Kolmogorov-Smirnov/Shapiro-Wilk test’. Descriptive analyses of normally distributed variables were indicated as mean differences, 95% confidence interval (95% CI), means (X) and standard deviation (SD); median and interquartile range (IQR) values were indicated for non-normally distributed variables, as well as percentage (%) and frequency (n) for categorical variables. Student-t test was used to compare normally distributed variables, and Mann-Whitney U and Chi-square tests were used for undistributed and categorical values. The level of significance was determined as p < 0.05.

## RESULTS

**Figure 1:**
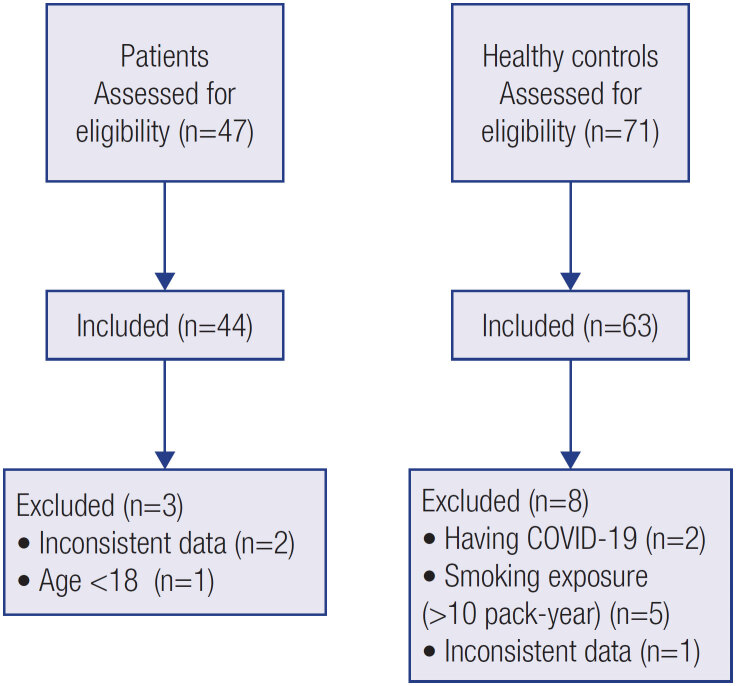
Flow diagram of patients with type 1 diabetes mellitus and healthy controls.

A total of 118 individuals replied to the online survey. Finally, 44 patients (27.5 ± 7.12 years) and 63 controls (27.65 ± 7.16 years) were selected for analysis ( Figure 1 ). Demographic characteristics were similar except for smoking (p < 0.05). The demographic and clinical characteristics of patients were shown in Table 1 . Patients were all on intensive insulin therapy using multiple daily injections of 3 rapid acting and 1 long acting insulin or continuous subcutaneous insulin infusion with rapid acting insulin. Physical role limitations and general health perception subscale scores of SF-36 questionnaire in patients were statistically significantly higher compared with controls ( Table 2 , p < 0.05). Other parameters were similar in both groups (p > 0.05). Physical activity levels of participants were similar ( Table 2 , p > 0.05). However, 12 (28%) patients and 18 (29%) controls had low, 24 (56%) patients and 31 (50%) controls had moderate, 7 (16%) patients and 13 (21%) controls had high physical activity levels. Fifty (79.4%) controls and 29 (65.9%) patients were had moderate physical activity for less than 150 min/week. Fifty (79.4%) controls and 33 (75%) patients were had vigorous physical activity for less than 75 min/week. Anxiety and depression scores were similar in both groups ( Table 2 , p > 0.05). However, 20.5% of patients and 15.9% of controls had anxiety; 34.1% of patients and 34.9% of controls had depression. Eight (18.2%) patients’ subscale score of HFS-B, 7 (15.9%) patients’ subscale score of HFS-W and 9 (20.5%) patients’ total score of HFS was above 50%. The total score of HFS in patients was 28.32 ± 15.59 ( Table 1 ). ULS-8 scale scores were similar in both groups ( Table 2 , p > 0.05). The HbA1c levels of 65.1% of patients were above 7 mmol/mol.

**Table 1:** Demographic and clinical characteristics of patients with type 1 diabetes mellitus and healthy controls

Characteristics		Patients (n = 44) X ± SD/median (IQR)	Controls (n = 63) X ± SD/median (IQR)	p
Age, years		25.5 (22, 33.3)	25 (23, 30)	0.892
Male;female, n/%		11/25%; 33/75%	11/17.5%; 52/82.5%	0.342
Weight, kg		64.85 ± 13.41	64.22 ± 12.69	0.805
Height, cm		165 (160, 170)	166 (160, 172)	0.388
Body mass index, kg/m^2^		22.1 (20.6, 25.6)	22.1 (20.3, 25.5)	0.500
Smoking, pack-year		0 (0, 14)	0 (0, 9)	**0.0351^#^**
Smoking (current; ex-smoker; non), n/%		10/22.7%; 6/13.6%; 28/63.6%	6/9.5%; 4/6.3%; 53/84.1%	0.052**
Comorbidities				
	Nephropathy	2/4.5%		
	Neuropathy	3/6.8%		
	Retinopathy	1/2.3%		
Pre pandemic HbA1c (%)		7.64 ± 1.51		
Frequency of hypoglycemia (fr/week)		2.41		
Missed dose of insulin (fr/week)		0.45		
Total insulin dose (U/day)		41.51 ± 17.76		
Continuous subcutaneous insulin infusion (yes/no)		14; 31.8%/30; 68.2%		
Hypoglycemia Fear Survey (HFS)				
	HFS-B score (0-4)	1.32 ± 0.77		
	HFS-W score (0-4)	0.97 ± 0.78		
	HFS total score (%)	28.32 ± 15.59		

Abbreviations: fr, frequency; kg, kilogram; m, meter; mmol, millimolar; %, percent; U/day, units/day; SD, standard deviation.

Descriptive analyses were expressed using (X ± SD), median (IQR) and (n/%) for normally/non-normally distributed and categorical variables, respectively. Chi-square test **p < 0.05, Mann-Whitney U-test^#^p < 0.05.

**Table 2:** Comparison of physical activity levels, quality of life, anxiety, depression, loneliness perception in patients with type 1 diabetes mellitus and healthy controls

Parameters		(n=44) X ± SD/median (IQR)	Controls (n=63) X ± SD/median (IQR)	Means difference (95% CI) /U	p
IPAQ parameters					
	Total, MET-min/week	1194 (466, 1194)	1230 (522, 2043.8)	1308.5	**0.873**
	Moderate, MET-min/week	190 (0, 740)	160 (0, 480)	1224	**0.695**
	Vigorous, MET-min/week	0 (0, 640)	0 (0, 480)	1163	**0.376**
	Walking, MET-min/week	693 (99, 2772)	577.5 (66, 4158)	1230	**0.502**
	Sitting, h/d	6 (5, 8)	7 (4.9, 10)	1298	**0.670**
SF-36 parameters (0-100)					
	Physical functioning	95 (90, 100)	95 (90, 100)	1363	**0.878**
	Physical role limitations	75 (25, 100)	100 (75, 100)	1063,5	**0.021#**
	Emotional role limitations	66.67 (0, 100)	66.67 (0, 100)	1370	**0.915**
	Social functioning	62.50 (37.5, 100)	75 (50, 100)	1160	**0.145**
	Mental health	58.00 ± 19.13	60.83 ± 17.69	2.83 (-4.30 to 9.95)	**0.433**
	Vitality/energy	50 (40, 68.8)	60 (40, 70)	1250	**0.387**
	Bodily pain	90 (57.5, 100)	77.5 (67.5, 90)	1328	**0.712**
	General health perceptions	54.89 ± 21.74	67.22 ± 17.48	12.34 (4.80 to 19.87)	0.002*
HADS parameters (0-21)					
	Anxiety	7 (4.3, 10)	6 (4, 9)	1304	**0.602**
	Depression	5 (2, 9)	5 (2, 9)	1373	**0.934**
UCLA Scale (8-24)					
	Loneliness	12 (9, 17)	11 (10, 15)	1359.5	**0.866**

Abbreviations: d, day; h, hour; HADS, Hospital Anxiety and Depression Scale; IPAQ, International Physical Activity Questionnaire; MET, metabolic equivalent of task; min, minute; SF-36, Short Form-36; CI, confidence interval; SD, standard deviation.

Descriptive analyses were expressed using (X ± SD) and median (IQR) for normally/non-normally distributed variables, respectively. *p < 0.05 Student’s t-test, ^#^p < 0.05 Mann-Whitney U-test.

## DISCUSSION

On the first view, the most striking results of the present study are that role limitations because of physical problems is higher in patients with type 1 diabetes mellitus. In our study, physical activity levels of patients with type 1 diabetes mellitus are not different than controls during COVID-19 pandemic, although general health perception is worsened and fear of hypoglycemia are increased in patients with type 1 diabetes mellitus.

Lockdown measures have caused changes in the lifestyle of individuals ( 5 ). In the present study, we observed that the status of physical role and general health perception were negatively affected in patients with type 1 diabetes mellitus. Increased time spent at home and fear of COVID-19 may have caused physical role limitations and deteriorated general health perception in patients with type 1 diabetes mellitus. Quality of life in patients with diabetes mellitus is related to many factors such as aging, type of diabetes, complications, glycemic control, treatment regimen, psychosocial factors, duration of diabetes and demographic variables ( 25 , 26 ). Nunes-Silva and cols. reported lower scores for general health perception in patients with diabetes compared with controls before pandemic. In our study, despite pandemic, all mean scores of SF-36 parameters in patients were slightly higher than the results of Nunes-Silva and cols.’s results ( 27 ). Younger age of our patients and aforementioned factors associated with diabetes mellitus may result in this difference in the quality of life scores. Goldney and cols. concluded that quality of life of depressed diabetic patients is severely impaired in all domains of quality of life before the pandemic ( 28 ). In our study, although the anxiety and depression perception increased in the patients and controls, depression (34.1%) and anxiety (20.5%) accompanying diabetes mellitus may have worsened their two domains of quality of life. Future studies should investigate in detail the causes of worsening in quality of life during the pandemic in patients with type 1 diabetes mellitus.

Wegeberg and cols. reported that increased HbA1c is a predictor of deteriorated physical function and bodily pain scores. Furthermore, impaired glycemic control causes deteriorated quality of life ( 29 ). Verma and cols. found that glycemic control has worsened in patients with type 1 diabetes mellitus during the lockdown period compared to the pre lockdown period ( 30 ). In our study, according to results of HbA1c evaluated before the pandemic, patients commonly had impaired glycemic control. Unfortunately, HbA1c results were not available in the pandemic. Increased HbA1c levels may cause increased diabetic complications ( 31 ). Therefore, there is a need for future studies to investigate the effects of glycemic control on diabetic complications and quality of life in the pandemic. Previous studies emphasize that multidisciplinary team care approach improves quality of life and mental health parameters of patients with diabetes ( 32 , 33 ). The virtual multidisciplinary approach is of great importance in the management of diabetes in the COVID-19 pandemic ( 33 ).

COVID-19 pandemic has caused lifestyle changes and decreased physical activity and increased sedentary time even in healthy individuals ( 34 ). Both patients and controls of our study had dramatically reduced physical activity levels and decreased moderate-vigorous physical activity intensity, and increased sedentary time in the pandemic. Furthermore, most of the patients (65.9%;75%) and controls (79.4%;79.4%) were not able to meet the criteria for moderate and vigorous physical activity levels required to maintain cardiovascular health recommended by the WHO ( 35 ). Lockdown has decreased physical activity habits in patients with type 1 diabetes mellitus ( 36 ). According to Falkowski and cols. ( 37 ) total physical activity scores of patients with type 1 diabetes mellitus and healthy individuals were higher before the pandemic when compared with our patients and controls. Ammar and cols. reported that mean MET values of the total, vigorous, moderate physical activity intensity and walking before lockdown were seriously is declined and sedentary time increased in healthy population compared to results during quarantine in the healthy population ( 38 ). In our study conducted in the pandemic, physical activity levels in controls were more affected compared to the results of Ammar and cols. both before and during confinement. COVID-19 pandemic and restrictions might have deteriorated physical activity levels not only in patients but also in healthy controls in the current study. A two week decline in daily steps from ∼10,000 to ∼1,500 steps causes deteriorated insulin sensitivity and lipid metabolism ( 39 ). Therefore, it is important to maintain physical activity levels and initiate physical activity counseling for all individuals during the pandemic.

COVID-19 has also triggers mental health problems such as anxiety, depression, and post-traumatic stress symptoms ( 40 ). The results of a recent meta-analysis ( 40 ) which showed the prevalence of anxiety at 31.9% and prevalence of depression at 33.7% during pandemic are in concordance with results of our control group. Before the pandemic Maia and cols. reported that 13.6% of patients with type 1 diabetes mellitus had depression and 16.4% had anxiety ( 41 ). In our study the ratio of anxiety (20.5%) and depression (34.1%) of patients during the pandemic is higher than Maia and cols.’s study. On the other hand in our study, mental health of controls was also negatively affected. Mental health may be worsened during COVID-19 pandemic in individuals. In such cases, telerehabilitation, which improves glucose control, exercise capacity and psychosocial status in patients with diabetes mellitus, can be considered ( 42 ) as telerehabilitation may be effective in healing mental health and improving functional capacity in patients with type 1 diabetes mellitus.

Severe fear of hypoglycemia leads to poor glycemic control and inadequate diabetes management behavior in patients with diabetes mellitus. Previous studies emphasized that fear of hypoglycemia is associated with severe hypoglycemia and personality factors like anxiety ( 43 , 44 ). Like previous study’s ( 44 ) survey findings, the results of current study were indicated that anxiety related to hypoglycemia and avoidance behaviors due to hypoglycemia risk increased. In our study, patients suffering from substantial fear of hypoglycemia (20.5%) was in considerable majority compatible with Nixon and Pickup’s results (27%) obtained from the evaluation of the fear of hypoglycemia in patients type 1 diabetes mellitus ( 21 ). However, unlike other studies ( 43 , 44 ) Nixon and Pickup ( 21 ) found that fear of hypoglycemia was not associated with HbA1c and explained that it was affected by more other factors. A recent systematic review emphasized that fear of hypoglycemia was common problem and caused impaired quality of life in patients with diabetes ( 45 ). Studies investigating fear of hypoglycemia in adult population were conducted before the pandemic ( 43 , 44 ). This study is the first to evaluate the fear of hypoglycemia in patients with type 1 diabetes mellitus during pandemic. Fear of hypoglycemia of patients may be related to many factors caused by the pandemic such as lockdown, anxiety, and loneliness. Thus, approaches to reduce the fear of hypoglycemia in patients should be developed during the COVID-19 pandemic.

Physical activity levels of patients decrease due to diabetes-related complications and they may have problems in adapting to social life ( 46 ). Maintaining social distance, avoiding handshakes, hugs, fear of the COVID-19 lead to changes in social interactions and contribute to loneliness perception ( 47 ). The current study showed that loneliness perception similarly increased in both groups. Diabetes-related complications and lockdown because of COVID-19 may have led to loneliness perception in patients. Furthermore, increased loneliness perception of controls may also be related to lockdown and changing lifestyle.

HbA1c of patients could not be measured during the pandemic due to the fear of catching COVID-19 in the hospital. This study was planned in pandemic period so there are no data about patients’ pre-pandemic physical activity levels, quality of life, anxiety, depression and loneliness perception.

In conclusıons, mandatory changes in daily lifestyle due to COVID-19 pandemic importantly affected not only patients but also controls. Crucial parameters of quality of life in patients with type 1 diabetes mellitus have deteriorated compared to controls. Individuals were had low physical activity levels. In this sense, telerehabilitation ( 42 ) can be considered as a method to increase physical activity, improve the quality of life, decrease anxiety, depression and loneliness perception for both patients and controls who can not participate in outpatient rehabilitation program during the pandemic. The importance of a multidisciplinary approach in diabetes management and dealing with problems should be considered in pandemic.
